# When Our Work Hits Home: Trauma and Mental Disorders in Correctional Officers and Other Correctional Workers

**DOI:** 10.3389/fpsyt.2020.493391

**Published:** 2021-02-15

**Authors:** Nina Fusco, Rosemary Ricciardelli, Laleh Jamshidi, R. Nicholas Carleton, Nigel Barnim, Zoe Hilton, Dianne Groll

**Affiliations:** ^1^Integrated Forensic Program, Ontario Provincial Police, Ottawa, ON, Canada; ^2^Department of Sociology, Memorial University of Newfoundland, St. John's, NL, Canada; ^3^Department of Psychology, University of Regina, Regina, SK, Canada; ^4^Department of Psychiatry, Queen's University at Kingston, Kingston, ON, Canada; ^5^Waypoint Centre for Mental Health Care, Penetanguishene, ON, Canada; ^6^Department of Psychiatry, University of Toronto, Toronto, ON, Canada

**Keywords:** therapeutic alliance, responsivity, prison, trauma, forensic employees, correctional employees, wellbeing

## Abstract

**Background:** International estimates suggest that up to one in three public safety personnel experience one or more mental disorders, including post-traumatic stress disorder (PTSD). Canadian data have been sparse until very recently, and correctional officers and forensic psychiatric staff have rarely been included. Working as a correctional officer is associated with negative health outcomes and increased work-related stress, with several variables affecting reported levels of stress. Healthcare staff also report higher rates of PTSD, especially those who are exposed to aggression in their workplace. In the present study, we compare current symptoms of diverse staff working in correctional occupations.

**Method:** Data were collected from a Canadian national online survey of public safety personnel, including employees of correctional services at the federal level. Correctional officers and wellness services staff were compared for prevalence of mental disorders and suicidal ideation.

**Results:** Correctional officers self-reported statistically significantly more exposure to potentially psychologically traumatic events than wellness services employees. Correctional officers also self-reported higher rates of symptoms of mental disorders, including PTSD, social anxiety, panic disorder, and depression. There were no statistically significant differences in reports of suicidal thoughts, plans, or attempts.

**Contribution to Society:** Correctional and forensic staff contribute to society by working with justice-involved individuals in correctional institutions. Trauma-related disorders and other mental health problems threaten the well-being of correctional and forensic staff. Mental health likely impacts the ability of correctional and forensic staff to develop a therapeutic or working alliance with persons in custody. Staff well-being must be recognized and addressed to ensure that prisoners and staff receive optimal treatment in prison.

**Conclusion:** Our results add to the limited knowledge about the well-being of staff, particularly wellness staff in prisons, who provide daily treatment and care for prisoners with serious mental disorders. Our work is a step toward identifying avenues for promoting staff well-being.

## Introduction

The past decade has seen an increased focus on research examining exposure to potentially psychologically traumatic events [PPTEs; (1)] among military personnel and first responders such as police, fire fighters, and paramedics [e.g., (2–4)]. Public safety personnel [PSP; (1, 5)], including communications officials, correctional workers, firefighters, paramedics, and police officers, are exposed to PPTEs by the very nature of their work (6). The potentially adverse effects of PPTE exposures in PSP workplaces were underscored by Mitchell (7). Critical incidents have been defined as line of duty experiences that provoke uncommonly strong adverse reactions (1, 7). Similarly, the phrase operational stress injury [OSI; (1)] was coined by Canadian military personnel to clarify the potentially significant negative mental health impacts of events experienced or witnessed in the line of duty (5, 8). Relatedly, burnout, vicarious trauma, and compassion fatigue are all terms that have been used to describe various aspects of workplace exposure to negative events (1, 9).

Estimates from the general North American population suggest that up to 90% of people are exposed to PPTEs, including the unexpected death of a loved one (10); however, only 5–10% of the general population will meet criteria for post-traumatic stress disorder [PTSD; (10–12)]. Criteria for PTSD according to the fifth edition of the Diagnostic and Statistical Manual of Mental Disorders (DSM-5) include experiencing, witnessing, or indirect exposure to one or more PPTE, as well as experiencing intrusion symptoms, avoidance, negative alterations in cognitions or mood, and alterations in arousal and reactivity (13). PSP are unique in their increased and heterogeneous exposure to PPTEs as part of their regular work duties (6) and report wide-ranging responses to PPTEs (14). Increased exposure to trauma alone may not account for the variability in reactions to PPTEs. The nature, severity, and frequency of PPTE exposures, as well as occupation type and occupational stressors, have all been associated with diverse rates of PTSD (2, 6, 15). In a recent study, the most common event identified by communications officials, correctional workers, paramedics, and police officers as being the worst event they had ever experienced was sudden violent death (6, 16).

International estimates suggest that up to one in three PSP meet diagnostic criteria for a mental disorder [e.g., (2–4)]. In a recent nationwide Canadian study 44.5% of PSP reported having significant clusters of symptoms consistent with at least one mental disorder. The most common mental disorders identified by screening measures were PTSD (23.2%) and major depressive disorder [26.4%; (14)]. Rates of mental disorders among PSP were consistently higher than diagnostic rates in the general population [i.e., 10.1%; (17)]. A separate Canadian study evidenced that reported rates of some mental disorders (i.e., PTSD, generalized anxiety disorder, panic disorder, social anxiety disorder) correlated positively with the number of exposures to different PPTEs types (6). Overall, PSP appear to be at increased risk for PTSD and several other mental disorders when compared to the general population.

Institutional correctional workers face a complex and unique set of challenges as a result of their confined workplace spaces and their daily interactions with incarcerated individuals (18, 19). A United States Department of Justice report listed several of the dangers and risks faced by correctional officers, including work-related dangers (e.g., prisoners with infectious diseases or mental illness, gangs, disruptive behavior), institution-related dangers (e.g., role ambiguity/conflict, inadequate resources, poor leadership/trust), psychosocial dangers (e.g., media/political scrutiny), mental health risks (e.g., stress, burnout), and physical health risks [e.g., injuries, death; (20)]. Accordingly, there appears to be ample evidence that correctional workers, and correctional officers in particular, work in an environment where concerns for safety can be omnipresent and the physical conditions are often inadequate or poor (21). In the United States, individuals working in prisons reported experiencing an average of 28 events of violence, injury, or death, and being a victim of an average of two assaults throughout their careers (22). Such statistics often fail to include verbal and sexual harassment, which is disproportionately experienced by correctional staff (23).

Correctional workers with less involvement in decision-making, poor job satisfaction, and decreased commitment to their work have reported higher levels of job-related stress (24). Indeed, the extent to which correctional officers exercise their power may play a role in shaping the institutional culture (25). Dowden and Tellier (24) found evidence that perceptions of danger were also associated with job-related stress, but variables such as shift work, security level of the institution, and years of experience were not significantly related to stress. Instead, officers with a human service or rehabilitation orientation toward prisoners reported considerably less stress than officers who endorsed statements reflecting a custodial or punitive stance. Australian correctional officers appear to perceive their work environment as more threatening and unpredictable than their counterparts working in general occupations (26). Australian correctional officers also appear more likely to experience PPTEs at work and report heightened levels of vigilance and caution with their actions that their counterparts working in general occupations (26).

The difficult work conditions experienced by correctional workers have been associated with adverse health outcomes, increased work-related stress, and other negative life events (15, 18, 19, 26). Correctional officers report significant rates of depression, physical health problems (stress-related illness, heart attacks, blood pressure, ulcers), burnout, compassion fatigue, work-home conflict, divorce, and even a shortened life span (6, 24, 27, 28). Correctional workers appear to have high levels of emotional exhaustion, depersonalization, and burnout (29, 30). The mental health challenges, as well as physical exhaustion and disengagement, among officers appear related to alcohol consumption (31). Exacerbating factors for mental health challenges among correctional workers include self-identifying as a woman, living alone, experiencing a range of PPTEs, and experiencing a large number of PPTEs (32).

Regarding PTSD specifically, a study of 3,599 correctional workers in the United States found rates of PTSD to be 27% (22). Individuals meeting the diagnostic threshold for PTSD experienced a greater number and variety of PPTEs (resulting in violence, injury, or death) and had experienced more types of assaults than those who screened negative for PTSD. Spinaris et al. (22) also found that correctional workers screening positive for PTSD demonstrated statistically significant higher frequencies of memory impairment, depression, sleep difficulties, digestive problems, heart disease, skin conditions, and obesity than those screening negative.

Nursing staff and other healthcare professionals report significant mental health challenges (33) and are also employed in correctional settings; accordingly, healthcare professionals work in the same difficult work settings as correctional officers. Healthcare staff are not typically included in groups of first responders or PSP; nevertheless, researchers have started to examine work-related mental health issues among institutional and community healthcare employees. Nurses report frequent PPTE exposures (33), with evidence that psychiatric nurses report high rates of exposure to violence and other disturbing patient behaviors (34). For example, there is evidence that most healthcare employees working in emergency departments (83.7%) report having experienced violence (34), with a mean of 28.22 such events in their careers; almost 40% of nurses, slightly more than half of direct care providers, and more than 20% of physicians report experiencing workplace violence. A meta-analysis of studies published between 1995 and 2014 of violence in psychiatric inpatient units further evidences that healthcare workers are routinely exposed to workplace violence (35). Iozzino et al. (35), for example, found 17% of patients on inpatient psychiatric wards displayed violence during their hospital stay.

Healthcare professionals also report elevated rates of PTSD, particularly those exposed to aggression at work (36, 37). An estimated 10% of health services employees report experiencing symptoms of PTSD, but that figure increases to 23% for nurses (33) and to 61% for those exposed to workplace violence (38). Hilton et al. (37) found that 24% of health services employees in a Canadian psychiatric hospital reported meeting the cut-off on a screening measure for symptoms of PTSD. Rates were higher for exposed nursing staff (31%) than for other allied health professionals (11%). Nursing staff working in psychiatric hospitals were also significantly more likely to have experienced disturbing patient behavior, such as hoarding, drinking toilet water, constant screaming, and smearing feces (37). PTSD symptom scores also appear related to the number of disturbing patient behaviors experienced by health services employees (37). In a survey of staff at three psychiatric hospitals, PTSD symptoms were associated with disturbing patient behaviors as much as with critical events, such as violence and threats (39). Forensic staff, who provide care for individuals charged with criminal offenses, reported exposure to more violence and threats than non-forensic staff, and were twice as likely to meet a screening cut off for PTSD (33). Thus, healthcare staff working with justice-involved individuals in the forensic system may be at increased risk for experiencing PTSD symptoms.

Correctional officers and other wellness service providers exposed to aggression in the workplace appear to experience prevalent negative mental health outcomes; accordingly, in the present study we compared current symptoms of mental disorder reports from correctional officers to reports from correctional wellness service providers, all working in Canadian federal prisons. The duties of correctional officers and wellness staff differ, but both work together in the challenging environments with persons who have complex needs. The elevated rates of mental health symptoms amongst correctional officers [e.g., (14, 22, 28)], and healthcare professionals working in other settings [e.g., (33, 36)], suggest that rates would also be elevated among healthcare professionals working within correctional settings. Recognizing that correctional officers are more likely to be exposed to PPTEs than other correctional workers, we hypothesized that officers would report higher rates of symptoms of mental disorders, including PTSD, depression and anxiety disorders, than prison wellness staff and other prison staff (e.g., administrative, institutional governance, and programming staff). In addition, we hypothesized that correctional officers would report having more suicide-related thoughts and attempts than other correctional staff, with wellness staff reporting the lowest reported rates of suicidal ideation and attempts.

## Materials and Methods

In the current study, we analyzed a subset of the data collected from a cross-sectional survey of PSP in Canada. Details of the original data collection are described elsewhere (6, 14, 28); in brief, participation in the survey was solicited through emails to currently serving public safety personnel employed as correctional workers and officers, firefighters, paramedics, police officers, public safety communications officials (e.g., call center operators/dispatchers). Data were collected in English or French using web-based self-report survey methods. Responses were anonymous, and participants were provided with a link that they could use to go back to the survey and complete it over time if necessary. Invitations were sent via various participating public service organizations such as Public Safety Steering Committee (PSSC) of the Canadian Institute for Public Safety Research and Treatment (CIPSRT), in addition to numerous national and provincial PSP agencies.

The sample of correctional workers in the original survey was not limited to employees of federal correctional services; however, in the current study we limit our sample to PSP working as institutional correctional officers and in institutional wellness services.

Exposure to PPTEs in one's lifetime was captured in the Life Events Checklist for the DSM-5 (LEC-5). There were two items on the scale modified for contextual suitability given the frequency of some events for PSP: “natural disaster” was modified to “a life-threatening natural disaster,” and the word “serious” was added to “transportation accident.” Seven additional questions addressing PPTEs experienced in the workplace were also included. Participants were asked whether they had witnessed line of duty deaths; experienced or witnessed disasters/multiple casualty incidents; experienced or witnessed serious line of duty injuries; experienced or witnessed incidents involving the unusual or sudden death of children or harm of children; experienced, witnessed, or learned about the suicide of a close colleague or a superior; experienced or witnessed incidents that seriously threatened their life or the life of a colleague; or experienced or witnessed incidents where the victims were relatives or friends. The highest number participants could choose as a response option was 11 or more exposures and there were 17 types of PPTE presented; as such, the total number of event exposures was limited to a maximum of 187.

Indications of mental disorder(s) and symptom severity were assessed using the well-established self-report screening tools described below; however, screening tools alone are not diagnostic. A “positive screen” on any of the tools indicates that an individual has self-reported symptoms in a manner consistent with persons who have been diagnosed with a given disorder. Individuals would need to be evaluated by a trained clinician to determine diagnostically the presence or absence of a specific mental disorder.

PTSD was assessed using the PTSD Check List 5 [PCL-5; (40)]. The PCL-5 is a commonly used 20-item self-report tool that assesses the 20 symptoms of PTSD outlined in the DSM-5. Individuals are asked to rate how bothersome the 20 items are to them on a scale of 0 (not at all) to 4 (extremely). Participants were asked to choose one event that was the most distressing to them and rate the PCL-5 based on their selected event. A positive PTSD screen was indicated if the participant met minimum criteria for each PTSD cluster and exceeded the minimum clinical cut-off score of >32 on the PCL-5.

Major Depressive Disorder (MDD) symptoms were assessed using the Patient Health Questionnaire 9-item [PHQ-9; (41)]. The PHQ-9 asks individuals to consider the past 2 weeks and to rate nine symptoms of depression on a scale of 0 (not at all) to 3 (nearly every day). MDD is suggested if 5 of the 9 items are rated at least a 2 or 3, or if the two questionnaire items; “little interest or pleasure in doing things” and “feeling down, depressed or hopeless” are rated 2 or 3.

Panic Disorder (PD) symptoms were assessed using the Panic Disorders Symptoms Severity Scale [PDSS; (42)]. The PDSS is a seven-item severity scale where items are scored on a 5-point scale from 0 to 4. The measure was designed to rate the overall severity of PD symptoms, and a cutoff score of 9 or above is suggestive of a panic disorder.

General Anxiety Disorder (GAD) symptoms were assessed using the GAD 7-item Scale [GAD-7; (43)]. The GAD-7 is a seven-item questionnaire where individuals are asked to rate how often symptoms of anxiety, such as feeling nervous, anxious, or on edge, have bothered them on a scale of 0 (not at all) to 3 (nearly every day). Responses are summed and a cutoff score of 9 or above is suggestive of a GAD.

Social Anxiety Disorder (SAD) symptoms were assessed using the Social Interaction Phobia Scale [SIPS; (44)]. The SIPS is a 14-item measure of social anxiety symptoms that can be divided into three subscales of Social Interaction Anxiety, Fear of Overt Evaluation, and Fear of Attracting Attention. Subscale scores and an overall score were calculated and assessed in this study, and a SIPS total score of >20 was considered a positive screen suggestive of a social anxiety disorder.

Risky (hazardous) alcohol use was assessed with the Alcohol Use Disorders Identification Test [AUDIT; (45)]. The AUDIT is consistent with ICD-10 definitions of alcohol dependence and harmful alcohol use. The AUDIT is a 10-item list of questions relating to an individual's drinking behavior. Items are scored from 0 (no or never) to 4 (response depends on the question being asked). Responses are summed and a positive screen for risky alcohol use was a score >15.

The Depression, Anxiety, and Stress Scale-21 (DASS-21) was also used to measure broad symptoms of depression, anxiety, and stress relative to general population data (46). The DASS-21 items are scored from 0 (does not apply to me at all) to 3 (applies to me very much or most of the time) and summed for each subscale (depression, anxiety, stress). Unlike the previous self-report measures, the DASS-21 is not a screening tool, but a measure of symptom severity.

Participants reported their symptoms in the timeframe per the instructions for each scale: PCL-5, past month; MDD, past 14 days; PDSS, past 7 days; GAD-7, past 14 days; SIPS, currently, no specific time window; AUDIT, past year; and DASS-21, past 7 days. Participants were also asked to report on lifetime suicidal ideation, and attempts using a series of yes/no questions intentionally aligned with precedent suicide items from Statistics Canada (17, 47). Suicidal ideation was assessed by asking, “Have you ever contemplated suicide?” and suicide attempts were assessed by asking, “Have you ever attempted suicide?”

All statistical analyses were conducted using SPSS Version 26 software. Demographic characteristics for both correctional officers and wellness staff are reported using frequency counts and percentages. Age, positive screens on each mental illness inventory, suicide-related variables, and trauma-related variables were compared between corrections officers and wellness services using Mann-Whitney *U*-tests. Logistic regression, controlling for sex, number of years working, and exposure to PPTEs was performed for each diagnostic screening test for each occupational group separately, as well as for group membership. The percentage of missing values across mental disorders (i.e., PCL-5, PHQ-9, GAD-7, SIPS, PDSS, AUDIT, and DASS-21 indicating more severe symptoms of depression, anxiety, and stress) varied between 2.1 and 21.5%. According to Little's Missing Completely at Random (MCAR) test, data appeared to be missing at random [$\chi_{\left(101\right)}^{2}$ = 75.27, *p* = 0.974]. Missing data were treated as missing (i.e., not imputed) because there was a sufficient sample size to perform the analyses and all statistical tests were considered significant at *p* ≤ 0.05.

## Results

There were 5,813 participants who completed the survey^1^. There were 1,308 respondents who were categorized as “correctional workers” and we limited our current analyses to the subset of 427 respondents who were employed either as institutional correctional officers (*n* = 359) or in institutional wellness services (*n* = 68) and at least responded to one of the mental disorder tools. “Institutional correctional officers” included institutional correctional, parole, and security intelligence officers, while “institutional wellness services” included nurses, psychologists, behavioral counselors, social workers and occupational therapists. The participants were primarily working in medium (33.5%), multi-level men's (24.1%), or maximum (19.2%) security institutions. Other institutions were minimum, multi-level women's, special handling unit, and a healing lodge (16.6%). From the excluded respondents, 216 were employed either as institutional correctional officers or institutional wellness services who did not respond to any mental disorder tools and 665 worked in roles outside of institutions. They worked either in community corrections or in administrative (national or regional headquarters) correctional roles, and as such were outside of the defined study population.

The total sample was split nearly in half by sex; specifically, 51.8% of respondents self-identified as male and 47.5% as female. There were statistically significantly more females in the wellness services group (72.1%) than in the correctional officers group (43.3%) (*p* < 0.001). The distribution for other sociodemographic variables are presented in Table 1.

**Table 1:** Sociodemographic variables between institutional wellness services and corrections officers.

**Sociodemographic variables**	**Correctional officers**		**Wellness services**	
	***n***	**%**	***n***	**%**
**Sex**				
Male	202	56.7	19	27.9
Female	154	43.3	49	72.1
**Age**				
18–29	15	4.2	1	8.8
30–39	105	26.0	18	30.9
40–49	130	36.3	22	32.4
50–59	93	29.3	21	30.9
60 and older	15	4.2	6	8.8
**Marital status**				
Married/Common-law	252	70.8	47	70.1
Single	37	10.4	10	14.9
Separated/Divorced/Widowed	41	11.5	8	11.9
Re-married	26	7.3	2	3.0
**Province of residence**				
Western Canada (BC, AB, SK, MB)	192	54.5	30	44.1
Eastern Canada (ON, QC)	123	34.9	31	45.6
Atlantic Canada (PEI, NS, NB, NFL)	37	10.5	7	10.3
**Ethnicity**				
White	309	86.1	56	82.4
Other	50	13.9	12	17.6
**Education**				
High school or less	47	13.7	–	–
Some post-secondary (<4-year college/university program)	159	46.2	20	29.9
University degree/4-year college or higher	138	40.1	47	70.1
**Language first spoken**				
English	294	82.1	41	60.3
French	56	15.6	19	27.9
Other	8	2.2	8	11.8
**Years of service**				
More than 16 years	196	54.6	31	45.6
10–15 years	84	23.4	16	23.5
4–9 years	70	19.5	17	25.0
<4 years	9	2.5	4	5.9

Regarding PPTEs, correctional officers reported more event exposures as assessed by the LEC-5 than wellness services employees, but the difference was not statistically significant. Almost all of the items related to workplace PPTE exposures were statistically significantly greater for correctional officers than for wellness services employees. Correctional officers had reported statistically significantly more exposures to the following: witnessing line of duty deaths experiencing or witnessing disasters/multiple casualty incidents; experiencing or witnessing serious line of duty injuries; experiencing, witnessing or learning about the suicide of a close colleague or a superior; and experiencing or witnessing incidents that seriously threatened their life or the life of a colleague (see Table 2).

**Table 2:** PPTE-related comparisons between institutional wellness services and corrections officers.

	**Corrections officers**	**Wellness services**	***p*-value**	**Effect size *(d)***
Trauma Exposure (LEC-5); Mean number of exposures (SD)	38.3 (28.5)	31.7 (25.4)	0.079	0.24
Witnessed line of duty deaths.	163 (45.4)	22 (32.4)	0.047^*^	0.20
Experienced or witnessed serious line of duty injuries.	280 (78.0)	33 (48.5)	<0.001^***^	0.52
Experienced or witnessed disasters/multiple casualty incidents.	136 (37.9)	19 (27.9)	0.118	0.16
Experienced or witnessed incidents involving the unusual or sudden death of children or harm of children.	51 (14.2)	12 (17.6)	0.464	0.08
Experienced or witnessed incidents that seriously threatened my life or the life of a colleague.	249 (69.4)	32 (47.1)	<0.001^***^	0.35
Experienced or witnessed incidents where the victims were relatives or friends.	132 (36.8)	22 (32.4)	0.487	0.06
Experienced witnessed or learned about the suicide of a close colleague or a superior.	234 (65.2)	36 (52.9)	0.055	0.18

* *p < 0.05*.

** *p < 0.01*.

*** *p < 0.001*.

*Unless otherwise indicated data are represented as n (%) of individuals responding “yes”*.

A higher proportion of women worked as wellness services (72.1%) compared to correctional officers (43.3%; see Table 3). Statistically significant differences were observed for PCL-5, PHQ-9, depression, anxiety, stress, SIPS and PDSS between wellness services and corrections officers. More specifically, positive screening of these mental health disorders was more prevalent among correctional officers compared to wellness services (see Table 3). Logistic regression analysis controlling for sex, years of employment, and exposure to PPTEs showed correctional officers are statistically significantly more likely to have screened positive on the SIPS (AOR = 3.49, 95% CI [1.33, 9.16], *p* < 0.05), the PCL-5 (AOR = 2.08, 95% CI [1.02, 4.27], *p* < 0.05), the PDSS (AOR = 3.93, 95% CI [1.17, 13.20], *p* < 0.05), the PHQ-9 (AOR = 2.09, 95% CI [1.05, 4.14], *p* < 0.05) than wellness services employees; however, there were no statistically significant differences in positive screens between groups on the GAD-7 or the AUDIT.

**Table 3:** Demographic and screening comparisons between institutional wellness services and corrections officers.

	**Wellness services**	**Corrections officers**	***p*-value**	**Effect size (*d*)**
Sex (% Female)	49 (72.1)	154 (43.3)	≤0.001^***^	0.43
**Suicide**				
Previous contemplation	27 (40.3)	111 (32.2)	0.046	0.20
Contemplation prev. 12 months	5 (4.5)	37 (7.0)	0.139	0.26
Prev. suicide plan	18 (16.2)	60 (11.3)	0.420	0.14
Prev. suicide attempt	6 (5.4)	19 (3.6)	0.972	0
PTSD (PCL-5)	11 (17.2)	115 (32.6)	0.014^*^	0.24
Depression (PHQ-9)	14 (21.2)	126 (36.5)	0.016^*^	0
Depression (DASS21)	9.10 (7.4)	11.82 (8.6)	0.032^*^	0.27
Anxiety (DASS21)	8.90 (7.7)	11.51 (8.7)	0.048^*^	0.26
Stress (DASS21)	8.24 (7.4)	11.79 (8.4)	<0.001^***^	0.35
Generalized Anxiety (GAD-7)	11 (16.9)	88 (26.5)	0.103	0.16
Social Anxiety Disorder (SIPS)	6 (9.5)	77 (23.9)	0.011^*^	0.26
Panic Disorder (PDSS)	3 (4.9)	59 (18.7)	0.008^**^	0.28
Alcohol Use Disorder (AUDIT)	1 (2.0)	30 (10.6)	0.051	0.22

* *p < 0.05*.

** *p < 0.01*.

*** *p < 0.001*.

*Data are represented as n (%) of respondents answering yes, DASS21 data are represented as mean (standard deviation)*.

Logistic regression analyses were conducted with positive mental health screenings derived from the mental health measures (i.e., PDSS, GAD-7, PHQ-9, PCL-5, AUDIT, and SIPS) for each occupational group while controlling for sex, years of work, and the number of exposures to PPTEs. The results indicated that, in correctional staff only, there was a statistically significant association with the number of PPTEs and positive screening on the PCL-5 (AOR = 1.02, 95% CI [1.01, 1.02], *p* < 0.001), PHQ-9 (AOR = 1.02, 95% CI [1.01, 1.02], *p* < 0.001), GAD-7 (AOR = 1.02, 95% CI [1.01, 1.03], *p* < 0.001), and PDSS (AOR = 1.01, 95% CI [1.01, 0.97], *p* < 0.01). There were no statistically significant associations among the wellness staff.

## Discussion

Correctional and wellness staff working in correctional settings contribute to society by working with justice-involved individuals in correctional institutions. Their work is invaluable in ensuring the safety, care, and custody of individuals who are incarcerated. In studies examining the larger data set from which the data for the current study were taken, exposure to PPTEs appeared to increase the risk of both PPTE-related disorders and other mental health problems for PSP (6, 14). The nature of correctional environments places staff working in correctional settings at a high risk for PPTE exposures, which poses a threat to their mental health and well-being.

In the present study, correctional officers and wellness services employees all reported extremely frequent exposures to workplace PPTE. Correctional officers (32.6%) and wellness services employees (17.2%) both screened positive for PTSD at higher rates than identified for the general population [i.e., 9.2%; (48)]. Correctional officers and wellness services employees also both reported more difficulties with suicidal thoughts (40.3 and 32.2%, respectively), plans (16.2 and 11.3%, respectively), and attempts (5.4 and 3.6%, respectively) than the general population [i.e., 11.8; 4; and 3.1%, respectively; (49)].

When the relationship between exposure to PPTEs and scores on screening measures for mental disorders was examined, only correctional officers' scores on a measure of PTSD symptoms was positively correlated with their exposure to PPTEs. This may be due to the nature of incidents rather than the number of exposures. As noted above, correctional officers were significantly more likely to have witnessed deaths in the line of duty; experienced or witnessed disasters/multiple casualty incidents, serious line of duty injuries, and incidents that seriously threatened one's life or the life of a colleague; and experienced, witnessed, or learned about the suicide of a close colleague or a superior. The exposure to more serious traumatic events may account for the correlation between the LEC-5 exposure and screening positive for PTSD for correctional officers but not for wellness services staff.

The results are generally consistent with previous results for correctional workers from the current sample (6, 14, 16, 28). The current paper provides novel and important results indicating significant and substantial differences between correctional officers and wellness services employees. Correctional officers reported significantly more frequent exposures to workplace PPTE than wellness services employees. Correctional officers were more likely than wellness services employees to witness deaths in the line of duty; experience or witness serious line-of-duty injuries; experience or witness incidents that seriously threatened their life or the life of one of their colleagues. Correctional officers were also significantly more likely than wellness services employees to screen positive for PTSD, SAD, and PD, but not for risky alcohol use, or for suicidal ideation, planning, or attempts. Correctional officers were not significantly more likely to screen positive for GAD or MDD than wellness services employees, but had significantly higher scores on the DASS-21 indicating more severe symptoms of depression, anxiety, and stress.

The differences between correctional officers and wellness services employees may be due, in part, to their specific vocational duties. Correctional officers have both security and wellbeing functions while healthcare staff are the very people who provide daily treatment and care for people with serious mental disorders in prison (50). The effectiveness of their work, or the ability for workers to create relationships and provide support for those in their custody, is very much tied to two interconnected phenomena: (i) the wellbeing of the staff; and (ii) staff ability to connect with and build rapport with those in their care. Responsivity and the ability to build and maintain a therapeutic alliance between care recipient and provider can be impaired if the care provider is also struggling with compromised mental health (51).

Correctional officers reported significantly more difficulties with mental health, but wellness services employees were still reporting more difficulties than the general population and would also benefit from additional research and support. Correctional officers and wellness services employees work interactively, getting to know their colleagues and the persons in their shared custody. As such, responding to calls for crisis intervention for officers means responding to the call for help of an individual (or individuals) they know personally—possibly a friend. Such responses add a layer of complexity to workplace PPTE exposures experienced by correctional workers.

An added dimension that is unique to those working in correctional settings is the conflict inherent at the crossroads of custody, care, and control. Correctional health professionals must provide services while upholding rules that sometimes interfere with their ability to provide care (52). Both correctional officers and those whose duties are to provide wellness services to prisoners must balance their mandate to confine with their inherent tendency toward caring for others (18, 52). Bell et al. (32) found variable and sometimes contradictory levels of organizational and peer support, whereby many mental health nurses reported that they were often or always supported regarding the emotional demands of their job and consulted on changes at work, yet most correctional officers reported little or no support or consultation. All staff reported feeling supported by their peers (32). These distinctions between organizational and peer support may help further explain differences between correctional officers and those working in wellness service roles in correctional settings.

The current results highlight the need for initiatives that promote the wellbeing of all correctional workers, including access to services focused on managing PTSD. Promoting well-being could assist with recruitment and retention of correctional workers, a group with tremendously high turnover rates (30, 53). The same aspects that attract helping professionals to work with populations with complex needs, such as incarcerated individuals, may also negatively affect wellness service employees (54). There is no literature that specifically examines the link between symptoms of mental disorders, such as PTSD, and the provision of services in correctional settings; nevertheless, mitigating the impact of PPTE on correctional staff will likely support maximizing the care provided.

People exposed to workplace PPTE and who work with populations that have complex needs (e.g., correctional officers, prison wellness staff) appear susceptible to compassion fatigue and burnout (55). Factors associated with compassion fatigue would inevitably affect the care provided to incarcerated populations, including poor judgment, apathy, desire to quit, lack of energy, unresponsiveness, callousness, and indifference (56). Research on the interaction between staff culture, power, and prisoner quality of life suggests that there may also be an important relationship between the culture and environment in correctional institutions and quality of life for prisoners (25). Accordingly, staff perceptions and their capacity to exercise authority appear associated with the institutional atmosphere and may play a role in the mental health of correctional staff.

Correctional psychologists appear to have lower job satisfaction than psychologists working in counseling centers, and higher rates of burnout than psychologists working in both counseling centers and for veterans' affairs (57). Thus, high turnover rates among correctional psychologists are not surprising, but could perhaps be reduced if more can be done to promote staff well-being and provide support (58). There is no direct evidence demonstrating exactly how the work of correctional workers and correctional officers in particular is impacted by PTSD; however, the literature on compassion fatigue and burnout would suggest that incarcerated individuals are adversely affected when service providers' ability to compassionately fulfill their roles is compromised.

The current study is limited in that only workers with access to organizational email responded to the study, and data from individuals on sick leave with limited or no access to the survey were unavailable. We recommend future researchers recruit staff on leave as participants, or staff who have left the organization completely, to understand how their mental health has been affected by their work. We were unable to determine whether there were any differences between staff that opted to participate in the survey and those that did not. Survey participants may not accurately represent the entire population of correctional workers; as such, there may be a self-selection bias (e.g., overrepresentation of those who were motivated to participate because of their views that their work contributes to poor outcomes or alternatively overrepresentation of those who have better outcomes because of higher energy, concentration, or motivation levels). The results are also limited because the data are cross-sectional and retrospective self-report. Future researchers could use longitudinal interview data to further contextualize the current results. The small proportion of staff working in each health-related profession means wellness services employees were grouped together rather than assessed in subgroups by profession (e.g., social work, psychology); accordingly, we could not analyze data in smaller subgroups within health services without potentially compromising respondent anonymity. Future researchers could extend the sample to be able to explore the nuances between different mental health symptoms among wellness staff as well as between different levels of correctional officers (e.g., those who work with male prisoners or primary care workers who work exclusively with female prisoners). Investigating the extent to which exposure to stressful or critical incidents account for variation in the presence of symptoms would also shed further light in the area. We asked participants to report on lifetime experiences of PPTEs to understand the total burden of PPTE exposures among correctional workers; however, participants would have reported on exposures (e.g., those involving natural disasters, children, relatives) that did not occur in the workplace. Future research that separates workplace and non-work exposures, or focuses on recent exposures, may be valuable for describing the workplace characteristics. Finally, results examining the relationship between total number of PPTE exposures and mental health sequalae may be limited by a ceiling effect because the maximum number of exposures was limited to 187.

The potential impact on correctional workers from mental disorder symptoms resulting from PPTE exposure appears significant. Mental disorders impact functioning and can have deleterious effects on work performance (59, 60), including capacity to develop a therapeutic or working alliance with persons in custody. Accordingly, action must be taken to address those suffering from symptoms in order to maximize their ability to adequately perform their duties. Increasing awareness through promotion and education about trauma and mental health, increasing access to trauma-informed supports such as peer support and advocacy programs, dedicated healthcare professionals for correctional workers, and increasing insurance benefits for psychological and other mental health services in the community, are all ways that might help improve overall well-being for correctional staff. Institutional correctional workers must also be supported in balancing their compassion with necessary boundaries for their own self-care to ensure sustainability at work and at home (23). Ultimately, ensuring the mental health and well-being of all correctional workers is critical not only for the workers, but for the diverse populations they serve.

## Data Availability Statement

The datasets generated for this study will not be made publicly available. The data analyzed in this study was obtained from CIPSRT, and access is restricted to CIPSRT researchers as specified in the use of data license agreement between CIPSRT and the various Public Safety Unions and organizations involved in the study.

## Ethics Statement

The studies involving human participants were reviewed and approved by University of Regina Institutional Research Ethics Board (File #2016-107). The patients/participants provided their written informed consent to participate in this study.

## Author Contributions

RR, DG, NF, and RC contributed conception and design of the study. DG, NB, RR, and NF organized the database. NF, DG, and NB performed the statistical analysis. NF, RR, DG, and NB wrote the first draft of the manuscript. NF, RR, NB, ZH, and RC wrote sections of the manuscript. All authors contributed to manuscript revision, read, and approved the submitted version.

## Conflict of Interest

The authors declare that the research was conducted in the absence of any commercial or financial relationships that could be construed as a potential conflict of interest.

## Footnotes

^1^Tracking the exact number of surveys distributed was not possible because of the recruitment processes across the PSP community, which allowed for and encouraged recipients to forward the invitation to others.
